# Factors Associated with Children’s Subjective Wellbeing During COVID-19 Pandemic in Bangladesh

**DOI:** 10.1007/s12187-023-10044-y

**Published:** 2023-06-15

**Authors:** Haridhan Goswami, Gour Gobinda Goswami, Bijoy Krishna Banik, M. Ibrahim Khalil

**Affiliations:** 1 Department of Sociology, Manchester Metropolitan University, Manchester, UK; 2 Department of Economics, North South University, Dhaka, Bangladesh; 3 Department of Sociology, University of Rajshahi, Rajshahi, Bangladesh; 4 Department of Sociology, Govt. Brojomohun College, Barishal, Bangladesh

**Keywords:** Children, Wellbeing, Happiness, Bangladesh, South Asia, COVID-19, School, Inequality

## Abstract

The study of subjective wellbeing has received increasing interest among social science researchers and policy makers in the wake of the COVID-19 pandemic. However, there appears to be a gap in the knowledge in terms of how the children experienced the pandemic, which may be different from the experience encountered by the adults. This article fills this gap by (a) examining children’s self-reported experience of the pandemic in Bangladesh and (b) identifying the socio-demographic, economic and psycho-social factors which were associated with their subjective wellbeing during the pandemic in the country. For this purpose, a child friendly questionnaire was developed, and a survey was conducted among 1370 children aged 10–12 years. The disruption caused by the pandemic was evident in children’s reporting of the movement of families from their usual place of living, job losses by their parents, food poverty, digital divide, and fear of the pandemic. In terms of factors affecting children’s wellbeing, eight factors (rural-urban differential, food poverty, digital inequality, support from friends and family, ability to manage learning from home, self-perceived safety, and worry about changes in student life) were found to have had statistically significant association with their wellbeing during the pandemic. These findings are discussed in the context of child wellbeing theories and previous empirical studies. Some policies are identified and put forward as recommendations for improving children’s wellbeing in Bangladesh.

## Introduction

Promoting children’s wellbeing is vital for children to have a good childhood and is the basis for their future wellbeing as adults (Rees et al., 2012). The aspiration to secure the wellbeing of children is explicit in the Grand Challenges such as the UN’s Sustainable Development Goals (SDGs). Among the 169 targets attached to them, 95 are either directly (48) or indirectly (47) connected to children. The SDGs can only deliver on the promise of equity if the world knows which children and families are thriving and which are being left behind (UNICEF, 2017). However, more recently countries are facing enormous challenges to meet those targets because of the COVID-19 pandemic affecting billons of children globally.

The world’s response to the COVID-19 pandemic focused initially on the biomedical emergency and this was reflected in the unprecedented amount of financial aid and investment to support medical research, equipment, vaccines, and treatments. Later, governments introduced specific policies to support children and families. While some of these policies have supported millions of children and families, COVID-19 is not the first and will not be the last virus to threaten humanity (Clark et al., 2020).

Although medical research has advanced very well to prevent deaths and find a cure for the virus, research assessing the social and psychological impact of the pandemic is still limited. Some of these aspects are examined narrowly among adults, however, systematic research among children regarding their experience during the pandemic is still limited. Children as active agents/stakeholders should be at the forefront of debates around the impact of COVID-19 pandemic on wellbeing and they should play a key role in shaping the debates and co-creating appropriate policy measures to mitigate the effect of the pandemic. Guided by this principle, this research was carried out in Bangladesh in collaboration with Children’s Worlds under its *COVID-19 Special Survey*. This article presents results from that survey.

The first aim of this article is to examine children’s self-reported experience of the pandemic in Bangladesh by focusing on (a) whether their families moved, (b) the economic aspects of their own lives and their families, and (c) the psycho-social aspects of their lives, in particular the level of support that they received from friends, and family, their ability to manage learning from home, self-perceived safety, and their worries about changes in student life.

The second aim of this article is to identify the socio-demographic, economic and psycho-social factors of the pandemic which are associated with children’s subjective wellbeing.

Findings from this research will be vital for policy makers to formulate child-centred and evidence-based policies for improving children’s wellbeing in the post-pandemic phase. The study will also extend the current knowledge base regarding the impact of the COVID-19 pandemic on children’s lives.

The rest of the article is structured in the following way: Section 2 presents a conceptual framework by defining the concept of children’s subjective wellbeing. Section 3 includes a review of key studies linked to children’s wellbeing and the influence of COVID-19 pandemic on children’s lives. It then presents a regression model on children’s wellbeing that is tested in this article. Section 4 discusses the methods used in this research. Section 5 presents the results of the study while, Section 6 discusses the key findings in the context of previous empirical studies and theories on subjective wellbeing. Section 7 draws some policy measures for improving children’s wellbeing in the post pandemic recovery phase. Section 8 concludes with acknowledging some limitations and putting forward some suggestions for future studies.

## Children’s Subjective Wellbeing: a Conceptual Framework

The concept of ‘wellbeing’ has been used in many different contexts. It is, therefore, essential to explain how the concept is used in the present research. In academic literature, it is used as an over-arching concept to refer to the quality of life of people in society (Rees et al., 2010a). Despite substantial academic and policy interest in wellbeing over the decades, there is no universally accepted definition of the concept. In defining it, a distinction is usually made between the hedonic and eudaimonic approaches (Ryan & Deci, 2001). The hedonic approach views wellbeing in terms of subjective happiness and the experience of pleasure versus displeasure, broadly constructed to include all judgements about the good/bad elements of life. The eudaimonic approach maintains that not all desires — not all outcomes that a person might value – would yield wellbeing when achieved (Ryan & Deci, 2001). It focuses on meaning and self-realisation and defines wellbeing in terms of the degree to which a person is fully functioning. Ryff and Keyes (1995) spoke of psychological wellbeing (PWB) and presented a multidimensional approach to the measurement of PWB that consists of six distinct dimensions: autonomy, personal growth, self-acceptance, life purpose, mastery, and positive relatedness. Although there is much debate among the followers of these two approaches, evidence from several investigators (e.g., Biswas-Diener et al., 2009; Proctor et al., 2014) has indicated that wellbeing is probably best conceived as a multidimensional phenomenon that includes both hedonic and eudaimonic elements.

For this article, wellbeing is defined by focusing on the hedonic perspective. In this article, children’s subjective wellbeing refers to “children’s evaluations of their lives – the degree to which their thoughtful appraisals and affective reactions indicate that their lives are desirable and proceeding well” (Diener et al., 2015: 234). See Section 4.2.4 for more information on how the subjective wellbeing scale is developed to measure the concept of wellbeing in the present article.

## Literature Review

### Factors Associated with Children’s Wellbeing

There has been a growing interest among researchers in children’s subjective wellbeing (Ben-Arieh, 2008; Ben-Arieh et al., 2001; Bradshaw et al., 2007; Cummins & Lau, 2005; Fattore et al., 2007; Casas, 2011; Huebner, 1991a; Konu et al., 2002; McAuley et al., 2012; McAuley & Layte, 2012) over the last few decades. Influenced by bottom-up theories (Diener, 1984), researchers have emphasised situational factors in explaining variations in subjective wellbeing. Among the situational factors, socio-demographic factors including age, gender, disabilities, learning difficulties, ethnic background, religious affiliation, family structure and family economic conditions. appear to draw a lot of attention among researchers (Rees et al., 2010b, 2012; Huebner, 1991b).

In recent years, researchers appear to focus on psychological factors in explaining variation in young people’s—mainly older group’s—wellbeing. Studies show that SWB among adolescents is associated with self-esteem (Dew & Huebner, 1994; Mowrer & Parker, 2004) and internal locus of control (Ash & Huebner, 2002; Dew & Huebner, 1994). Recent research containing both sociodemographic and personality characteristics appeared to explain 33.5% of the variation in children’s subjective wellbeing (Goswami, 2012). Researchers also found that children’s experience of being bullied as a key factor for their wellbeing (Borualogo & Casas, 2023). Children’s relationships with family, friends, and their self-perceived safety are also important factors for their wellbeing (Rees et al., 2010a, b). Although these studies have played vital role in identifying important factors which are linked to children’s subjective wellbeing, more studies are needed to understand how these factors influenced children’s wellbeing during the COVID-19 pandemic.

### COVID-19 Pandemic and Wellbeing

The literature on COVID-19 and its far-reaching impact can be broadly classified into three types. The first type deals with the transmission, its reasons, and severity through the general holiday or government intervention through lockdown (Goswami et al., 2021a; Goswami et al., 2021b). The second type of study deals with its impact on inequality, stock market contagion, and other effects (Goswami & Labiba, 2021). The third type deals with the geo-economics, social and political aspects of the pandemic (Barai & Dhar, 2021). Based on the findings from some early research, Coyne et al. (2020) argue that the effects of COVID-19 on families vary widely depending on contextual stressors parents experience. Research from the US found disparities in terms of morbidity across different racial and ethnic groups, with black and ethnic communities suffering higher rates of COVID-19 illnesses and deaths (Oppel et al., 2020). Moreover, higher infection among lower income families suggests that families in communities under the greatest social and economic burdens have been placed under conditions such that they are more vulnerable to becoming sick (Valentino-DeVries et al., 2020). Daly and Robinson (2021) looked at some nationally representative studies in the UK and US and found that mental health deteriorated immediately following the onset of the COVID-19 pandemic (Daly et al., 2020; Li & Wang, 2020; McGinty et al., 2020; Pierce et al., 2020). The rise in mental distress has been attributed to pandemic-related stressors including risk of infection and death, financial concerns, and enforced isolation (Robinson & Daly, 2020).

In the UK, Wolfe and Patel (2021) have looked at the Understanding Society’s COVID-19 survey to examine how self-employment during the pandemic is related to financial worries. Their findings indicate that self-employed individuals experience greater financial worries during the pandemic and that this increase in financial worries corresponded with a rise in mental distress for the self-employed. Coyne et al. (2020) argue that the COVID-19 pandemic has confronted many parents with difficult choices. While some parents with key worker roles were dealing with the stress of going back and forth to work and the potential contamination of their homes, others (working from home) are dealing with increased demands of homeschooling their children while still trying to meet their own employment requirements. These demands are further increased for parents of children with developmental delays, chronic emotional or behavioural difficulties, or other health challenges. As demands and parent stress increase and resources dwindle, children may also be placed in increased proximity of domestic abuse (Tolan, 2020).

As Spinelli et al. (2020) argue, the majority of studies conducted during previous pandemics and from the beginning of the COVID-19 outbreak tended to examine psychological consequences on the general population, leaving the study of effects on parents and children mainly unexplored, with few exceptions (Brooks et al., 2020) which looked at the impact on children’s educational aspects focusing narrowly on drop out, attendance, and performances. Moreover, the few studies which looked at children’s wellbeing during the COVID-19 pandemic were conducted predominantly in  Western and/or economically developed countries. While these studies have made significant contribution to our understanding on the impact of the pandemic on children’s lives, we know very little about the pandemic’s influence on children’s subjective wellbeing in Bangladesh, which has around 64 million children. This article fills this gap by using Children’s Worlds Special Survey on COVID-19 pandemic completed in 2021.

Based on the review of these literature, we developed and tested a regression model on children’s subjective wellbeing during the COVID-19 pandemic by incorporating three broad types of factors: (a) *socio-demographic* (age, gender, area of living, moving home during the pandemic); (b) *economic* (parental job loss, food poverty, digital inequality); (c) *psycho-social factors* (support from friends, support from family, managing learning from home, self-perceived safety, and worries about changes in student life).

#### Regression Model

Children’s subjective wellbeing during the pandemic.

1 $$\begin{array}{l}{SubWell}_i=\beta_0+\beta_1 \ {\text{Gender}}_i+\beta_2 \ {\text{Age}}_i+{\beta_3 \ \text{Rural}}_i+\beta_4 \ {\text{Moving home}}_i+ \\ \beta_5 \ {\text{Parental job loss}}_i+\beta_6 \ {\text{Food poverty}}_i+\beta_7 \ {\text{Digital inequality}}_i+\beta_8 \ {\text{Friend support}}_i+ \\ \beta_9 \ {\text{Family support}}_i+\beta_{10} \ {\text{Managing home learning}}_i+\beta_{11} \ {\text{Self}-\text{perceived safety}}_i+ \\ \beta_{12} \ {\text{Student life worry}}_i+\varepsilon_i \dots \end{array}$$

The subscript i: 1, 2, 3…, 1124 (cross-section dimension representing each respondent). ‘SubWell’ represents ‘subjective wellbeing,’ considered as the dependent variable. Gender, age, rural, moving home, parental job loss, food poverty, digital inequality, friend support, family support, managing home learning, feeling safe, and student life worry are considered as independent variables in the model. ε represents the random error term which captures all the omitted variables, functional misspecification, and measurement errors in determining the dependent variable. It is assumed that the error term follows a normal distribution which facilitates us in conducting regular T-tests and F-tests. The expected sign of the dummy coefficients (linked to nominal level variables) bears a special meaning. For example, if found negative, the particular category has a lower dependent variable value than the reference category during COVID-19. The expected sign of regular coefficients indicates an increase if it is positive and a decrease if it turns out negative. The composite value of the dependent variable (life satisfaction scale) has wide variation within a broad range, allowing us to use the ordinary least squares (OLS) estimation method instead of logit or probit estimation. For full measurement description of the dependent and all independent variables, see Section 4.2 (measures).

## Methods

### Participants, Data Collection, and Ethics

For this study, data were collected from 1,370 children aged 10–12 years using two modes: face-to-face interview and online survey. In both cases, convenient sampling was used for which findings could not be generalized to the whole children population in Bangladesh. Bangladesh has 64 districts (administrative units). Interview data were gathered mainly by concentrating on three districts which were also included in previous wave (Wave 3) of Children’s Worlds survey in Bangladesh. These three districts were selected purposively for easy access to schools as the Co-Is in Bangladesh live in these three regions and they have easy access to schools in their own regions. Co-I from each region used their own professional networks and other links to find children in the three age groups. They have also contacted local school teachers for their support in obtaining access to children. Although convenient sampling was used, attention was paid to get a balanced representation of children by gender and rural-urban location (village, town/city) (Table 1).

**Table 1:** Distribution of sample by region and mode of data collection

Region	Data collection mode		Total
	Interviews	Online	
Barishal district (Southern part of Bangladesh)	350	0	350
Rajshahi district (North-West of Bangladesh)	360	0	360
Moulvibazar district (North-East of Bangladesh)	350	0	350
Dhaka city (and a few other regions)	0	310	310
Total	1060	310	1370

Originally, the plan was to gather data through face-to-face interviews only. However, when fieldwork was about to start, the Second Wave of COVID 19 hit Bangladesh and the country went under lockdown for a considerable period of time. It was not clear when the restrictions would be lifted. As there was a significant uncertainty on face-to-face data collection because of the lockdown, it was decided, data collection using online method during the lockdown period (2nd wave) should be commenced. However, almost two weeks after the launching of the online survey, lockdown restriction was gradually lifted although schools were still closed. For face-to-face interview, the survey team approached children mostly at their home. There were a few cases where children were interviewed in school setting when they came to collect homework from school on pre-defined dates by schools.

For online data collection, the Co-Is used their professional network (teaching) to find children of the selected age groups. Although there were some children who participated from other regions in the country, majority of the children who took part in the online survey came from large cities (mainly Dhaka—the capital city). The total number of children completing the online survey was 310 (22.6% of total sample in this study). Active consent was sought from both children and their parents or guardians before data collection started.

### Measures

#### Independent Variables: Socio-Demographic Factors

##### Gender

Children were asked to classify themselves either as boys or girls in the survey. Girls were kept as the focal category of interest and boys were dummy coded to 0 for advanced statistical analysis.

##### Age

In the survey, children were asked to report their age by choosing age from a set of pre-defined categories: 10, 11, and 12. Age was measured as a scale variable in this article.

##### Rural-Urban Residence

In the survey, children were asked to say whether they lived in a village or town/city. In this article, rural children were the focus of interest and were compared against urban children who were dummy coded to 0 for statistical analysis purpose.

##### Moving Home

The survey asked children to say whether their family had to move from their usual place of living because of the pandemic. Answers were gathered in Yes/No format. A dummy variable was created with code 1 for ‘Yes’ group and 0 for ‘No’ group.

#### Independent Variables: Economic Factors

##### Parental Job Loss

To measure the family’s economic hardship during the pandemic (Wang et al., 2021), the survey asked children to report whether either of their parents lost their jobs due to the pandemic and were not receiving any support from the government. Those children who reported their parents to have lost their job were given code 1 and those who did not report that experience were given code 0 for statistical analysis purpose.

##### Food Poverty

To measure the experience of living in poverty (Goswami et al., 2022), children were asked to report how often they had enough food to eat each day during the pandemic: always (coded 0), often (coded 1), sometimes (coded 2), and never (coded 3). This produced a single-item scale ranging from 0 to 3 (higher score indicating greater level of food poverty).

##### Digital Inequality

Digital inequality refers to the differences in the material, cultural and cognitive resources required to make good use of information and communication technology (OECD, 2015). To measure digital inequality, we asked children how often they had access to the Internet. Responses were captured under four categories: always (coded 0), often (coded 1), sometimes (coded 2), and never (coded 3). Using these code values, we created a single-item digital inequality scale. It ranges from 0 to 3 in which higher score indicates greater level of digital divide.

#### Independent Variables: Psycho-Social Factors

##### Support from Friends

Friends are regarded as a key source of support for children. To measure the level of support that they received from friends during the pandemic, we developed a five-point friendship support scale ranging from 0 (I do not agree) to 4 (Totally agree). It was developed from the children’s response to the following statement: ‘During the Coronavirus, I felt well-supported by some of my friends’.

##### Support from Family

To measure the level of support that children received from their parents or carers during the pandemic, they were asked to say how much they agreed with the following statement: ‘During the Coronavirus, I felt well-supported by some people I live with’. Children responded to this in a five-point Likert scale: 0 (I do not agree) to 4 (I totally agree). Higher the score, greater is the level of family support.

##### Managing Learning from Home

The survey provided a statement asking children to report their degree of agreement with the following: *During the Coronavirus, when schools were closed, I managed to continue with my learning from home*. Children provided response to this statement using a five-point response scale: 0 (I do not agree) to 4 (Totally agree). The higher the score in the scale indicates the greater the ability of children in managing learning from home during the pandemic.

##### Self-Perceived Safety

A five-point Likert scale was developed to measure children’s self-perceived safety during the pandemic. For this purpose, children were asked to say how much they agreed with the statement: I feel safe when I walk around in the area, I live in. For developing a 0–4 scale, their five-point responses were coded as follows: 0 (I do not agree), 1 (I agree a little), 2 (I agree somewhat), 3 (Agree a lot), and 4 (I totally agree). The higher the score in the scale indicates the greater level of self-perceived safety.

##### Worry About Student Life

Children’s worry about student life was measured by using an eleven-point rating scale ranging from 0 (Not at all) to 10 (Very much). In this regard, children responded to the following: During the last month, how worried have you been about the changes in your life as a student because of the Coronavirus situation? The higher the score in the scale indicates the greater level of worry about student life.

#### Dependent Variables: Subjective Wellbeing Scale

Over the past few decades, a number of measures have been developed for measuring subjective wellbeing. Three of which gained popularity are the single-item measure of happiness with life as a whole (Cummins & Lau, 2005), the single-item Cantril’s ladder (Cantril, 1965), the multiple-item life satisfaction scale (Huebner 1991a, b). Compared to the single item measure, the multiple-item measure of subjective wellbeing is reported to be more stable (Goswami, 2009). The Children’s Worlds have slightly modified the original scale of Hubner through several waves of its international survey through statistical testing (Casas & Rees, 2015). The items have been further modified in Wave 3 following discussions with children in low-income countries outside Europe with the aim of improving cross-cultural comparability. The final version of Children’s Worlds Subjective Wellbeing Scale (CW-SWBS) contains six items: (a) My life is going well, (b) I have a good life, (c) I like my life, (d) I am happy with my life, (e) I enjoy my life, and (f) The things that happen in my life are excellent. Children were asked to say how much they agree with each item on a scale from 0 (do not agree with the sentence at all) to 10 (agree with it completely).

A principal component analysis with orthogonal (varimax) rotation extracted one factor (total initial eigenvalue 4.07) explaining 67.87 per cent of the total variance. Therefore, these items are proved to measure a single construct of ‘Subjective wellbeing’. Internal consistency analysis of these six items obtained Cronbach’s Alpha of 0.90, which indicates excellent consistency of the scale. Score in each item was added together to create SWBS which ranges from zero to 60—a higher score indicating a greater level of wellbeing.

## Results

### Children’s Experience During the Pandemic

#### Socio-Demographic Characteristics of Children and Movement of Their Families During the Pandemic

Almost equal number of boys and girls participated in the survey (Table 2). Although the survey aimed to gather data from equal number of children from the three age groups, ten years old children (who usually study in class five—the final year in primary school) were slightly fewer than the other two age groups: 11-year-old who usually study in class six—the first year in high school and 12-year-old who usually study in class seven (second year) in high school. Furthermore, almost equal number of children took part from rural and urban areas. Approximately 4% of the children reported that their families had to move from their usual place of living because of the pandemic.

**Table 2:** Distribution of socio-demographic factors

Socio-demographic factors (N)	Percentages		
Gender (1353)			
Boy.	49.7		
Girl	50.3		
Age group (1353)			
10-years old	24.0		
11-years old	33.2		
12-years old	42.8		
Place of living (1353)			
Rural	49.1		
Urban	50.9		
Moving home during the pandemic (1322)			
Yes	4.2		
No	95.8		

#### Economic Aspects of Children’s Lives During the Pandemic

Families were under tremendous financial pressure during the pandemic. In the survey, almost one in five children reported that their parents lost their job because of the pandemic. The economic hardship was also reflected in food poverty. Almost one in five children reported their families to have enough food only ‘Sometimes’. Also, 6% of children reported that they ‘never’ had enough food to eat at home (Table 3). When these two response categories were merged, it was found that almost one quarter of children reported their families to have food insecurity during the pandemic.

**Table 3:** Distribution of economic factors

Economic factors (N)	Percentages	*Mean (sd.)*
Parental job loss (1209)		
Yes	19.4	
No	880.6	
Food poverty scale, 0–3 (1275) [Enough to eat every day]		0.7 (1.0)
Always (code 0)	58.7	
Often (code 1)	16.0	
Sometimes (code 2)	19.5	
Never (code 3)	5.7	
Digital inequality scale, 0–3 (1353) [Access to the Internet]		1.9 (1.1)
Always (code 0)	17.1	
Often (code 1)	17.6	
Sometimes (code 2)	24.4	
Never (code 3)	40.9	

Digital divide was also evident in the study. It appears from results in Table 3 that almost four out of ten children reported never having internet access and a further one-quarter reported to have internet access only sometimes. Therefore, overall, access to digital technology was very limited to many children during pandemic.

#### Psycho-Social Aspects of Children’s Lives During the Pandemic

To understand the nature and degree of support that children received during the pandemic, the survey asked questions on the level of support from family/carer, and friends. In this regard, over 7 out of 10 children felt well-supported (when ‘agree a lot’ and ‘totally agree’ categories were merged) by family/carer they lived with. However, children felt that they had less support from friends (almost 46% agreed little bit/did not agree) during the pandemic (Table 4). It is perhaps not surprising to see a high number of children reporting that way because their traditional platform—school where they meet friends—was closed for a very long time in Bangladesh during the pandemic.

**Table 4:** Distribution of psycho-social factors

Psycho-social factors (N)	Percentages	Mean (sd.)
Friend support scale, 0–4 (1353) [felt well-supported by friends]		1.7 (1.3)
I do not agree (coded 0)	22.8	
Agree a lit bit (code 1)	23.8	
Agree somewhat (coded 2)	25.0	
Agree a lot (coded 3)	14.3	
Totally agree (coded 4)	14.0	
Family support scale, 0–4 (1353) [felt well-supported by people I live with]		3.1 (1.1)
I do not agree (coded 0)	4.6	
Agree a lit bit (code 1)	6.1	
Agree somewhat (coded 2)	15.0	
Agree a lot (coded 3)	25.2	
Totally agree (coded 4)	49.1	
Managing learning scale, 0–4 (1340) [managed to continue learning from home]		2.5 (1.3)
I do not agree (coded 0)	11.3	
Agree a lit bit (code 1)	12.2	
Agree somewhat (coded 2)	24.7	
Agree a lot (coded 3)	19.3	
Totally agree (coded 4)	32.5	
Self-perceived safety, 0–4 (1353) [feeling safe walking around in the area]		2.0 (1.4)
I do not agree (coded 0)	21.1	
Agree a lit bit (code 1)	17.9	
Agree somewhat (coded 2)	24.2	
Agree a lot (coded 3)	16.6	
Totally agree (coded 4)	20.1	
Worry about student life, 0–10 scale		7.2 (3.3)

To understand home learning situation during the pandemic, the survey provided a statement asking children to report their degree of agreement with the following: *During the Coronavirus, when schools were closed, I managed to continue with my learning from home*. Table 4 presents results on a five-point response scale: totally agree, agree a lot, agree somewhat, agree a little, and do not agree. Whilst one-third of the children totally agreed with this statement, almost one in ten did not agree and further 12% only agreed a little bit with this. Therefore, the pandemic appeared to have a negative impact on children’s ability to learn from home.

In response to the question on their feelings on safety during the pandemic, around one-fifth (21.1%) of the children did not feel safe when they walked around in the area they lived. The survey asked children to express their degree of worry about  changes in life as a student due to the pandemic. Table 4 reports the mean score of 7.2 for children’s worry. As this score is much higher than the mid-point score of 5 in 0–10 scale, children’s worry about their future was found to be quite high in the study.

### Influence of Socio-Demographic, Economic, and Psycho-Social Factors on Children’s Subjective Well-Being During the Pandemic

Table 5 presents results of multiple linear regression analysis. It is noted that cases with missing values are excluded from the regression model. The statistically significant F value of 17.43 (*p* = 0.000) indicates that the overall subjective wellbeing model is statistically significant. The adjusted R^2^ value of 0.149 suggests that almost 15% of the variation in children’s subjective wellbeing during the pandemic was explained by those socio-demographic, economic, and psycho-social factors included in the regression model.

**Table 5:** Multiple linear regression analysis on children’s subjective wellbeing during COVID 19 pandemic by socio-demographic, economic, and psycho-social factors

	B	Std error	Standardized	t	Sig
			Beta		
Constant	32.39	7.02		4.61	0.000
Socio-demographic factors					
Gender (Male Ref.)					
Female	0.32	0.92	0.01	0.34	0.731
Age	-0.50	0.58	-0.02	-0.85	0.394
Living in rural area (No Ref.)					
Yes (in rural area)	2.94	1.03	0.09	2.86	0.004
Moved home (Ref. No)					
Yes	-3.91	2.40	-0.04	-1.62	0.104
Economic factors					
Parental job loss (Ref. No)					
Yes	0.79	1.20	0.02	0.66	0.510
Food poverty	-2.02	0.51	-0.12	-3.99	0.000
Digital inequality	-0.98	0.48	-0.07	-2.05	0.040
Psycho-social factors					
Support from friends	2.06	0.36	0.16	5.64	0.000
Support from family	1.89	0.43	0.13	4.38	0.000
Managing learning from home	2.11	0.36	0.17	5.78	0.000
Feeling safe	1.21	0.32	0.10	3.74	0.000
Worry about changes in student life	-0.30	0.11	-0.06	-2.10	0.036

Adjusted R^2^ = 0.149; F = 17.43; P = 0.000; N = 1124

Out of three socio-demographic factors included in the model, only area of living appeared to be associated with children’s subjective wellbeing during the COVID-19 pandemic. More specifically, children in the rural area (B = 2.94, *p* = 0.004) reported to have statistically significant higher level of satisfaction during the pandemic compared to those living in the urban area.

Among the three economic factors examined in the model, food poverty (B = -2.02, *p* = 0.000) and digital inequality (B = -0.98, 0.040) were significantly associated with children’s wellbeing. Children living under higher level of food poverty and digital inequality appeared to report significantly lower level of satisfaction in life during the pandemic.

Interestingly, all the five psycho-social factors were found to be associated significantly with children’s wellbeing during the pandemic. More specifically, higher level of satisfaction during the pandemic was reported by those children who received greater level of support from friends (B = 2.06, *p* = 0.000), family (B = 1.89, *p* = 0.000), and those who successfully managed learning from home (B = 2.11, *p* = 0.000), and felt safe during the pandemic (B = 1.21, *p* = 0.000). In this regard, higher degree of children’s worry about changes in student life during the pandemic appeared to be associated significantly with lower wellbeing (B = -0.30, *p* = 0.036).

When the relative impact of these eight factors is evaluated by comparing standardised beta values, it is observed that children’s ability to manage learning from home (Beta = 0.17) appeared to have the highest influence on children’s wellbeing during the pandemic. In this regard, the level of support children received from friends (Beta = 0.16) and family (Beta = 0.13) had the second and third highest impact on their wellbeing. Food poverty (Beta = -0.12) and feeling safe (Beta = 0.10) had respectively the fourth and fifth highest impact on children’s level of wellbeing during the pandemic. Although their impact was relatively smaller, living in rural areas (Beta = 0.09), and digital inequality (Beta = 0.07) also had respectively the sixth and seventh highest impact on children’s wellbeing during the COVID-19 pandemic. Although it was a significant factor, children’s worry about changes in student life appeared to have the lowest impact (Beta = -0.06) on their wellbeing.

## Discussion

The key aim of this article was to describe children’s experiences during COVID-19 pandemic and identify the socio-demographic, economic, and psycho-social factors which are associated with children’s subjective wellbeing during the pandemic in Bangladesh. This section discusses key findings from this research in the context of other empirical studies and theories on wellbeing.

The disruption in children’s lives caused by the pandemic was evident from children’s self-reported data. In some extreme cases, some families had to move from their regular place of living. Parental job loss was another dimension of the pandemic reported by the children. Food poverty and digital divide were two additional economic features of the pandemic that came to light from the children’s experiences.

In terms of the effect of the socio-demographic and economic, and psycho-social factors on children’s wellbeing, rural-urban differential appeared to play a significant role. Rural children reported to have higher level of subjective wellbeing during the pandemic compared to their counterpart. When this finding is examined in the context of previous studies, the influence of rural-urban differential on subjective wellbeing appears to be very inconclusive. Cummings et al. (2003) Knight and Gunatilaka (2010) found that rural residents had higher level of subjective wellbeing. However, other researchers such as Murray et al. (2004) and Millward and Spinney (2013), Goswami (2021) reported that urban residents had higher level of subjective wellbeing than their rural counterparts. Differential level of economic development in rural and urban areas is argued to be responsible for different degrees of happiness among residents in the two localities (Easterlin et al., 2011). Although in the context of Bangladesh, access to services and facilities – road infrastructure, electric power availability, healthcare, transportation, mobile phone signal strength – are generally limited to children living in rural areas, during the pandemic rural children were advantageous in certain aspects of their lives. They had better opportunities to utilize the open space available in their neighborhood for sports and other activities. This might be associated with higher subjective wellbeing for them.

In this research, almost one quarter of the children reported their families to have difficulty in accessing enough food for the members during the pandemic. Previous studies among adults found food insecurity to be associated with poorer mental health and specific psychosocial stressors across global regions independent of SES (Jones, 2017). Research among children (Goswami, 2021; Goswami et al., 2022) also found a statistically significant association between children’s experience of food poverty and lower level of subjective wellbeing. Jones (2017) explains this connection by emphasising several psychological dimensions of food insecurity. Firstly, by generating uncertainty over the ability to maintain food supplies, or to acquire sufficient food in the future, food insecurity can provoke a stress response that may contribute to anxiety and depression. Secondly, acquiring food in socially unacceptable ways can induce feelings of alienation, powerlessness, shame, and guilt that are associated with depression. Thirdly, food insecurity may also magnify socioeconomic disparities within households and communities that could increase cultural sensitivities and influence overall mental and subjective wellbeing.

This study found a significant number of children (four out of ten children) who never had internet access and a further one-quarter to have internet access only sometimes. Therefore, overall, access to this digital technology during the pandemic was very limited to many children. This has affected children’s educational wellbeing and relationship with friends as they could not access even those limited online educational services offered to them during the pandemic in Bangladesh because of not having the required devices and/or internet access. During the pandemic, use of technology increased dramatically in global context. Technology was used to manage the effects of the pandemic in many developed countries. Hutchings (2020) provides evidence on how the National Health Services (NHS) in the UK adopted digital technology for many of its services to free up space and capacity in acute hospitals, enable remote working and reduce the risk of infection transmission in NHS settings. In this regard, the Primary care saw a huge increase in remote appointments. The review of Vargo et al. (2021) documented systematically the use of digital technology in healthcare, education, work, and daily life during the pandemic. Their findings reveal that most of the educators and students choose to use video-based devices and platforms to continue their education to follow stay at home order. Although digital connectivity appears to be a lifeline to mitigate some of the effects of the pandemic, some studies found evidence of widening digital inequality between schools in deprived and affluent neighborhood within a country (Roberts, & Danechi, 2022) and also between the North and the South globally (Makau, 2021; Balfour et al., 2022).

Although the risk of exposure to COVID-19 was thought to be lower for children, compared to adults, children are more vulnerable to the emotional impact of traumatic events that disrupt their daily lives (Bartlett et al., 2020). In this study, children who reported to have higher level of support from family and friends were found to have greater level of subjective wellbeing during the pandemic. A previous study among children in England found that children’s relationships with friend had the second highest effect (after family relationship) on their overall wellbeing (Goswami, 2012). Researchers argue for a supportive network of people (family/carers, relatives, teachers, friends) to recover from a traumatic event. Morrow (2001) suggests that social networks promote a sense of belonging and wellbeing. There is evidence that life goals associated with a commitment to family, friends, social and political involvement promotes life satisfaction (Heady et al., 2011). Findings from these studies suggest that social relationships influence individuals’ psychological wellbeing by providing love, intimacy, and guidance.

In this study, higher subjective wellbeing was found to be associated with children’s greater ability to manage study from home. However, one of the biggest challenges for the children was to continue with their learning from home. The pandemic appeared to have negative impact on children’s ability to learn from home as over one out of five children reported to have difficulties studying from home when school was closed during the pandemic. The long-term effect of not being engaged with learning activity by children during the pandemic will have longer effect on their future aspiration, current learning, and future educational wellbeing. Although more studies are needed to capture the long-term effect of the pandemic on children’s education, currently available evidence suggests that the closure of schools and interruption with learning during the pandemic have impacted children in different ways, including delays in socio-emotional and behavioural development (Pearcey et al., 2020) and mental health, physical development (Bento & Dias, 2017) and school readiness (Nicholls et al., 2020; Tracey et al., 2022).

According to UNICEF, 37 million children in Bangladesh had their education disrupted by one of the world’s longest school closures (543 days) due to the pandemic. Although the Ministry of Primary and Mass Education (MoPME) and Ministry of Education (MoE) introduced remote learning through pre-recorded classes hosted on televised broadcasts and online platforms by the first week of April 2020 on a limited scale, the lack of online device and connections for remote learning appeared to be some of the key barriers to such initiatives. There is some evidence on the effect of inequalities in children’s experiences of home learning during the pandemic. Andrew et al. (2020) found considerable heterogeneity in children’s learning experiences - amount of time spent learning, activities undertaken during this time and availability of resources to support learning in the context of the UK. They found a strong association between this heterogeneity and family income. They argued that any impact of inequalities in the time spent on learning between poorer and richer children are likely to be compounded by inequalities not only in learning resources available at home, but also those provided by schools. Rahman and Sharma’s (2021) simulation model estimated the impact of school closure on students’ learning in Bangladesh. They argue that school closures will push more children into learning poverty. Their analysis shows that school closures will increase the share of children who do not attain the minimum reading proficiency at the end of primary (Grade 5) by 18% points to 76% and will affect children differently depending on their socio-economic characteristics.

Children’s assessment of how safe they feel is a key domain of their overall wellbeing (Rees et al., 2010a). Safety is a cross-cutting domain (Rees et al., 2010b)—with links among family, friends, school, and local area. Children who reported higher level of safety during the pandemic were found to have greater subjective wellbeing. Researchers have found that children’s perspectives on safety are different from those of adults (Côté-Lussier et al., 2014). Understanding children’s views on safety and identifying factors which are linked to this are crucial to develop evidence-based policies on children’s wellbeing. Different factors bring about variation in children’s assessment of safety and subjective wellbeing. In this regard, individual factors such as disability, learning difficulties, runaways, and being in out of care home appear to have influence on how children feel about their safety and rate their overall wellbeing (see Rees et al., 2010b for more information). Because of its complex connection with other domains and wellbeing, child safety was identified as one of the five outcomes for children and young people in Every Child Matters (DfES, 2003)—one of the key policy documents of the UK Govt. in 2003.

Overall, children’s level of worry regarding the impact of the pandemic especially their worry about the changes in student life was found to be very high. Some researchers e.g., MacLeod et al. (1991) argue that worry is a cognitive phenomenon affected by future events coming with uncertainty about potential outcomes, creating feelings of anxiety. There is evidence that worry serves to exacerbate anxiety (Dickson et al., 2012). Therefore, in most cases, children’s worries are expected to be associated with adverse outcomes, such as mental health developed after prolonged exposure to COVID pandemic.

## Policy Implications

This article demonstrates school children’s experiences of the COVID-19 pandemic in Bangladesh. The evidence came from children’s own assessment and evaluation of life during the pandemic. This section revisits the findings on key socio-demographic, economic, and psycho-social factors which were found to be associated with children’s subjective wellbeing during the pandemic and proposes a series of policy measures to improve children’s wellbeing in the post pandemic recovery phase.

### Access to Digital Technology

Digital technology played a vital role in overcoming some aspects of learning barriers during the pandemic in many countries. No access to internet (reported by around 60% children) and laptops and tablets when needed (reported by almost 73% children) was a major resource issue for these school children to continue with their learning when schools were closed during the pandemic. This inequality in digital technology needs to be addressed to level up learning gaps of these technology deprived children in Bangladesh.

### Financial Support to Poor Families

Almost one-quarter of the children in this study reported their families to have difficulty in securing enough food for families during the pandemic. These families need extra support to improve economic wellbeing in post pandemic recovery phase.

### Additional Support from Teachers

Bangladeshi children experienced one of the longest school closures during the pandemic. Although schools tried to provide some support to children through setting up homework for children, broadcasting pre-recorded lectures in radios and TVs for a limited period to make up some of the loses experienced by children from school closures during the pandemic, there is inequality among children depending on their family’s economic conditions in getting access to those limited supports provided by schools. Therefore, additional supports for their education and extra-curriculum activities should be in place for these children to boost their confidence on future prospect which was rated very low during the pandemic.

### Emotional Support

This study found a very high level of worry among children about the changes that happened to their lives as students from the pandemic. In addition to providing extra educational support, schools need to provide emotional support through counseling to the children so that they feel confident and supported with their learning and other aspects of their lives.

### Reducing Rural-Urban Differential

Children living in urban areas reported to have significantly lower wellbeing during the pandemic. Key factors linked to this differential need to be identified and actioned to improve the wellbeing of urban children.

### Ensuring a Safe and Supportive Network

In this research, level of support that children received from friends (Beta = 0.16) and family (Beta = 0.13) were found to have the second and third highest impact on children’s wellbeing. Feeling safe in the area of living was also a very important factor. Therefore, it is important to ensure a safe and supportive network around children to improve their overall wellbeing.

## Limitations and Future Directions

Despite many strengths, including a child friendly questionnaire, asking children directly about their experiences during the pandemic, good sample size, roughly equal representation of gender among the participants, the present study was limited by a number of factors.

Firstly, this study used convenient sampling for recruiting children. Moreover, children took part in the survey mainly from four regions. Therefore, the findings cannot be generalized to all children in Bangladesh. Future studies need to explore options for probability sampling and ensure participation of children from wider geographical regions.

Secondly, the study covered only a narrow age range (10–12) for children. Therefore, the study will not be able to capture age variation on children’s experiences robustly during the pandemic. Future studies need to widen the age bracket to assess how children’s experience (if at all) varies by age.

Thirdly, children were sampled only from mainstream schools for this research. In Bangladesh, different types of schools exist including faith-based schools, English medium. Therefore, this study could not capture what effects school types might have on subjective wellbeing. Future studies need to consider this aspect and include children from different types of schools.

Fourthly, since this study was conducted among mainstream schools, the sample did not cover some other groups of children such as children with disabilities, learning difficulties, street children and those who drop out and/or never went to school. Future studies need to consider those groups as well for a more comprehensive picture on factors associated with children’s subjective wellbeing during the pandemic in Bangladesh.

Fifthly, this study is based on cross-sectional design. Therefore, it does not provide evidence on causal links between pandemic and children’s wellbeing. Longitudinal data is needed to test and establish the causal links.

Finally, the regression model for children’s subjective wellbeing was developed by considering twelve factors out of which eight were statistically significant. They explained around 15% of the variation in the subjective wellbeing. Future studies need to improve this model by including other relevant psycho-social factors and their possible interaction effects.

## Data Availability

All data and materials including questionnaire are freely available. These are deposited to Children’s Worlds https://isciweb.org/ and researchers can get access to the materials by submitting their request to Children’s Worlds.

## Declarations

As part of submitting this article to Child Indicators Research Journals, the authors declare the followings:
